# Anatomic Landmarks of the Distal Radioulnar Joint

**Published:** 2015-08-14

**Authors:** J. Choo, A. Augenstein, M. Nadar, E. Afflick, M. Kasdan, B. Wilhelmi

**Affiliations:** ^a^Division of Plastic Surgery, University of Louisville, Louisville, Ky; ^b^Hunstad/Kortesis, Huntersville, NC; ^c^Department of Radiology, University of Virginia, Charlottesville; ^d^University of Louisville Medical School, Louisville, Ky; ^e^Department of Surgery, Veterans Affairs Hospital, Louisville, Ky

**Keywords:** distal radioulnar joint, ulnar-sided wrist pain, rheumatoid arthritis, steroid joint injection, palpation-guided joint injection

## Abstract

**Hypothesis:** Using Lister's tubercle and the ulnar styloid as landmarks, accurate localization of the distal radioulnar joint can be achieved without the need for an image-guided approach. **Methods:** Cadaveric dissection of 16 upper extremities was performed to measure the relationships between the ulnar styloid, Lister's tubercle, and the distal radioulnar joint. In each specimen, the location of the distal radioulnar joint (point A) in relation to Lister's tubercle and the ulnar styloid was determined as follows: (1) the perpendicular distance between the distal radioulnar joint and ulnar styloid–Lister's tubercle was measured; (2) with A' marking the intersection of this distance and the ulnar styloid–Lister's tubercle line, the location of the distal radioulnar joint along the ulnar styloid–Lister's tubercle axis was determined by comparing ulnar styloid–A' and A'–Lister's tubercle with ulnar styloid–Lister's tubercle. **Results:** The mean distance between ulnar styloid–Lister's tubercle was 4.3 ± 0.4 cm. The mean perpendicular distance between the distal radioulnar joint and the ulnar styloid–Lister's tubercle line was 0.2 ± 0.1 cm proximal to the ulnar styloid–Lister's tubercle line. The ratio of ulnar styloid–A' and A'–Lister's tubercle to ulnar styloid–Lister's tubercle was 0.5 ± 0.03 and 0.5 ± 0.03, respectively. **Conclusions:** Simple relationships between the ulnar styloid and Lister's tubercle serve as reliable landmarks for locating the distal radioulnar joint. The distal radioulnar joint is centered about the midpoint of the ulnar styloid–Lister's tubercle axis and slightly proximal to it. This may improve the accuracy and efficacy of corticosteroid injections in the treatment of distal radioulnar joint arthritis without the need for image guidance.

Steroid injections are often administered in the office for conditions of the hand and wrist, including basilar joint arthritis, carpal tunnel syndrome, De-Quervain's tenosynovitis, and trigger finger. Traditionally, injections for these conditions were performed by palpation, but with the advent of readily available imaging tools such as ultrasonography, image-guided injections have become more common. While the use of image guidance appears to improve the accuracy of injections, it is unclear from the current evidence whether image-guided injections improve patient-relevant outcomes to justify the added cost.^1^

Distal radioulnar joint (DRUJ) pain is a frequent cause of ulnar-sided wrist pain and is a common indication for steroid injection of the wrist. DRUJ arthritis has a number of causes, including accrued stress over a life time, traumatic injury, and rheumatologic conditions.^2^ Initial therapy for DRUJ pain usually consists of conservative therapies such as rest, nonsteroidal anti-inflammatory medications, static and/or dynamic splinting, and steroid injections.^3^ When combined with a local anesthetic, steroid injections have the added utility of helping clinicians diagnose the DRUJ as the site of ulnar-sided pain.^4^

Despite well-described techniques for successful DRUJ arthrography^4^ and ultrasound-guided DRUJ injections,^5^ no well-described landmarks for palpation-guided injections of the DRUJ exist in the literature. Because the average office setting may not have ready access to an ultrasound machine, a reliable and simple technique of steroid injections into the DRUJ by palpation is therefore necessary.

The authors report a cadaveric study that describes a straightforward technique for reliable intra-articular injection of steroid using readily palpable superficial landmarks: the ulnar styloid (US) and Lister's tubercle (LT).

## METHODS

**Figure 1:**
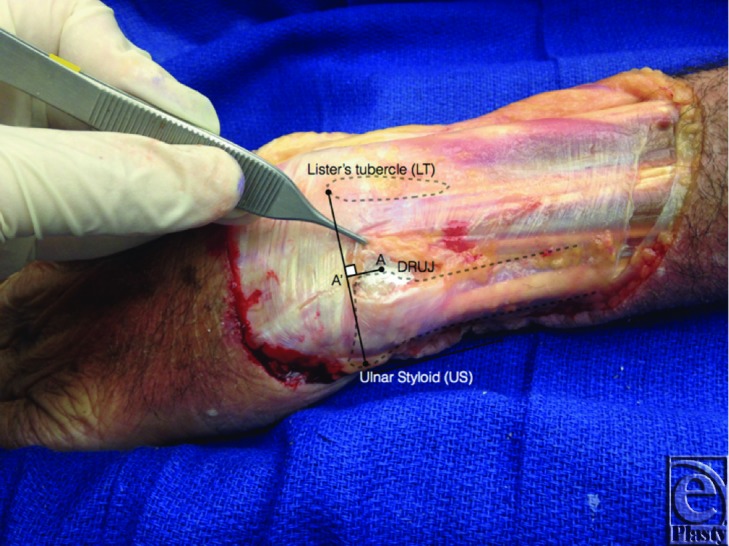
The DRUJ lies on a point approximately 2 mm proximal to the midpoint of the line connecting LT and US. DRUJ indicates distal radioulnar joint; LT, Lister's tubercle; and US, ulnar styloid.

Eight pairs of fresh cadaveric specimens (male: n = 4; female: n = 4) were used for the purposes of this study. The ages of the cadavers were not known. With the first 12 wrist specimens, LT and US were palpated and marked on the dorsal aspect of the wrist. The dorsal skin was raised as a flap and the intra-articular space of the DRUJ was marked at its midpoint (point A, Fig 1). With elbow bent at 90° and the forearm held in a neutral position (0° of pronation/supination), several measurements were then obtained (Table 1) using a caliper to 1 mm precision. The distance between LT and US (US-LT line) was measured using LT at its distal most aspect and the tip of US at its ulnar most point. The perpendicular distance from the DRUJ to the US-LT line was marked as the line connecting the DRUJ and the US-LT line at a perpendicular angle (A-A'). Point A' was defined as this intersection point on the US-LT line and was used to determine where the DRUJ falls along the US-LT line. The distance between US and A' and the distance between A' and LT was measured in relation to the overall US-LT distance for each specimen. The distances from US to A' and from A' to LT were measured. The ratios of US-A' to US-LT and A'-LT to US-LT were then calculated.

Next, 4 wrist specimens from male (n = 1) and female (n = 1) cadavers were injected on the basis of the measurements obtained to validate our findings. A 1:1 white latex/sterile saline mixture using a 25-gauge needle was injected at a point slightly ulnar to the midway point between the US and LT, with the needle tip angled radially and distally approximately 30° and 30°, respectively. The needle was advanced along the dorsal and radial surfaces of the ulna until firm resistance was met, at which point the needle was slightly withdrawn and 1 mL of latex/normal saline was injected. If firm resistance was met, the needle was slightly repositioned to allow injection. The dorsal soft tissue of the wrist was then raised as a flap to observe the location of the latex dye.

## RESULTS

On the basis of all 16 specimens, the mean distance between US-LT was 4.3 ± 0.4 cm. The mean perpendicular distance between the DRUJ and the US-LT line was 0.2 ± 0.1 cm proximal to the US-LT line. The ratios of US-A' to US-LT and A'-LT to US-LT were 0.51 ± 0.03 and 0.49 ± 0.03, respectively. From these data, the authors concluded that the DRUJ space lies just proximal to the midpoint of the US-LT line.

Latex dye injections based on these measurements revealed staining of the intra-articular space of the DRUJ in all cases. There was no staining of the extensor digiti minimi (EDM) or surrounding soft tissues.

## DISCUSSION

The DRUJ is a diarthrodial articulation between the distal radius and ulna that permits forearm supination and pronation via the rotation of the radiocarpal unit about the ulna. The head of the ulnar articulates with the sigmoid notch of the radius, but because of the size incongruity between the 2 articular surfaces, the bony articulation accounts for only 20% of stability of the DRUJ.^6^ Joint stability is mainly due to soft-tissue structures that form the anatomic boundaries of the joint space, as well as muscles that act as dynamic stabilizers. The boundaries of the DRUJ include the dorsal radioulnar ligament, the volar radioulnar ligament, and the triangular fibrocartilage complex, which account for much of the stability of the DRUJ.

The DRUJ can be involved in chronic pain after distal radius injuries, rheumatoid arthritis, and osteoarthritis. Consequently, the DRUJ is a frequent site of ulnar-sided wrist pain. Patients with arthritis involving the DRUJ complain of pain with pronation and supination, limited range of forearm rotation, clicking, crepitus, and decreased grip strength.

Management of DRUJ arthritis ranges from conservative to surgical. Initial therapy for symptomatic DRUJ arthritis consists of conservative therapies such as rest, nonsteroidal anti-inflammatory medications, static and/or dynamic splinting, and steroid injections.^3^ Steroid injections are best suited for symptomatic arthritis, with minimal radiographic findings, such as narrowing of the joint space, osteophytes, and other changes, can make accessing the joint space difficult and hamper the accuracy of clinically guided injections.^7^ Several studies have looked at the accuracy of clinically guided injections for osteoarthritis of various joints such as the trapeziometacarpal joint,^7^ the knee,^8^^,^^9^ the shoulder,^10^^,^^11^ and the elbow.^12^ What most of these studies demonstrated was that the accuracy of intra-articular injections guided by clinical examination was poor (29%–83%).^12^ Surprisingly, palpation-guided injection has been unreliable even in larger joints such as the shoulder and the knee,^10^^-^12 2 of the most common areas injected. In a study of 109 joint injections of the shoulder and the knee, only 56 were unequivocally intra-articular.^8^ While the accuracy of intra-articular placement does not necessarily correlate with symptom relief,^8^^,^^10^^,^^12^ the complications that can arise from inaccurate injections are cited by many authors as the rationale for the use of image guidance. These complications include the known local effects of steroids, such as soft-tissue atrophy,^8^ tendon atrophy, and rupture,^7^^,^^13^ as well as systemic effects, such as insulin resistance and hypertension, which have been shown to be more pronounced in soft-tissue than in intra-articular injections.^14^

Studies of small joints of the hand and wrist show that these joints, particularly when narrowed by disease, are difficult to enter reliably.^7^ In the case of the DRUJ, however, palpation-guided techniques for access to the DRUJ, both for wrist arthroscopy and arthrography, have been described.^15^ The volar and dorsal aspects of the DRUJ capsule have a redundancy that allows rotation of the radius about the ulna.^2^ Taking advantage of this redundancy, Lomasney and Cooper^15^ described a technique for positioning the needle slightly ulnar to the articular margin to reliably gain access to the intra-articular space of the DRUJ. Despite the fact that this study was performed with radiographic landmarks, the authors feel that the DRUJ is more amenable to palpation-guided techniques than other joints due to its superficial location and the presence of bony landmarks.

Structures at risk during a dorsal approach to the DRUJ include the transverse branches of the dorsal ulnar cutaneous nerve and the dorsal branch of the anterior interosseous nerve, which lie superficial to the DRUJ.^16^ Also at risk are the adjacent extensor tendons, particularly the EDM, which immediately overlies the intra-articular space. The EDM, along with the extensor digitorum communis to the ring and small fingers, is already at risk of attritional rupture in patients with DRUJ pain secondary to rheumatoid arthritis, and the consequent extension lag of the small and ring metacarpophalangeal joints (Vaughn-Jackson syndrome) may be one of the findings of this disease.

The use of steroid injections into the DRUJ in patients with rheumatoid arthritis may require more caution, but once a diagnosis of DRUJ arthritis or pain is made, the use of palpation-guided steroid injections for symptom relief should not necessarily be discouraged in favor of an image-guided approach. Our study shows that an accurate and consistent injection of the DRUJ space is possible with readily palpable landmarks on the dorsal wrist.

Limitations to this study include the fact that any relevant underlying pathology in these specimens, for example, prior fractures, existing DRUJ arthritis, could not be determined. Such information would have impacted the validity of our data but was not possible due to the inability to obtain prior medical records. Exacerbating this limitation is the lack of plain images for each specimen. This would have allowed detection of any pathology of the DRUJ or surrounding anatomy, as well as determine anatomic variants (ulnar variance) that may have affected our data. In addition, because this study was performed on cadavers, the clinical efficacy of injections could not be assessed.

The lack of a caliper with a higher degree of precision (eg, 0.1 mm) is also a limitation increasing the measurement error, given the small values of the perpendicular distance in each specimen and the fact that A-A' distance is 2 mm with a standard deviation of 1 mm. Inherent imprecision is at play when choosing point A, US, and LT as the basis of measurements. Nevertheless, these limitations do not affect the significance or basic conclusion of the study, which is that in the transverse plane the DRUJ can be found centered about the US-LT axis with a high degree of precision. First, the DRUJ is not a point but a joint that is longer than it is wide, with an average length of 7.5 mm on the radial side of the DRUJ and an average width of 1.6 mm.^17^ A point at the center of the DRUJ was chosen for measurement purposes. But the ultimate aim is to access the joint space, and even in the extremes of measurement of 0 to 4 mm found in our study, one would expect to be reasonably successful in accessing the DRUJ within its length using 2 mm proximal to US-LT line as a landmark. The location along the US-LT axis is the more important measurement in accessing the DRUJ, and the data here support that the DRUJ can be found with a high degree of precision at the midpoint of the US-LT line.

## Figures and Tables

**Table 1:** Measurements obtained from cadaveric specimens

Sex	Hand	US-LT, cm	A-A', cm	US-A', cm	A'-LT, cm	US-A'/US-LT	A'-LT/US-LT
F	R	4.1	0.3	1.9	2.2	0.49	0.51
F	L	3.5	0.2	1.9	1.6	0.50	0.50
F	R	3.9	0.4	1.9	2.0	0.46	0.54
F	L	4.2	0.3	2.1	2.1	0.54	0.46
F	R	3.9	0.3	2.1	1.9	0.53	0.47
F	L	4.3	0.1	2.2	2.2	0.50	0.50
F	R	4.2	0.3	2.1	2.1	0.50	0.50
F	L	3.9	0.3	2.0	1.9	0.51	0.49
M	R	4.3	0.2	2.5	1.8	0.58	0.42
M	L	4.0	0.2	2.0	2.0	0.50	0.50
M	R	5.0	0.0	2.5	2.5	0.49	0.51
M	L	5.0	0.0	2.5	2.5	0.48	0.52
M	R	4.7	0.1	2.3	2.4	0.50	0.50
M	L	4.8	0.1	2.3	2.5	0.50	0.50
M	R	4.2	0.4	2.2	2.0	0.52	0.48
M	L	4.0	0.4	2.0	2.0	0.50	0.50
	Mean	4.3	0.2	2.2	2.1	0.51	0.49
	SD	0.4	0.1	0.2	0.3	0.03	0.03

US indicates ulnar styloid; LT, Lister's tubercle.
